# Supplementary material for: Leveraging ancient DNA to uncover signals of natural selection in Europe lost due to admixture or drift
Source: Nat Commun. 2024 Nov 12;15:9772. doi: 10.1038/s41467-024-53852-8 (PMC11557891; doi:10.1038/s41467-024-53852-8)
Supplement: Supplementary file 1 — Supplementary Information [file 41467_2024_53852_MOESM1_ESM.pdf]

Leveraging ancient DNA to uncover signals of natural selection in Europe lost due to admixture or drift

Devansh Pandey[1†], Mariana Harris[2†] Nandita R. Garud[3, 4‡*], and Vagheesh M. Narasimhan[1, 5‡*]
[1]Department of Integrative Biology, The University of Texas at Austin, USA
[2]Department of Computational Medicine, University of California, Los Angeles, USA
[3]Department of Ecology and Evolutionary Biology, University of California, Los Angeles, USA
[4]Department of Human Genetics, University of California, Los Angeles, USA
[5]Department of Statistics and Data Science, The University of Texas at Austin, USA
† Equal Contribution
‡ Joint Supervision
* Co-correspondence: vagheesh@utexas.edu and ngarud@ucla.edu

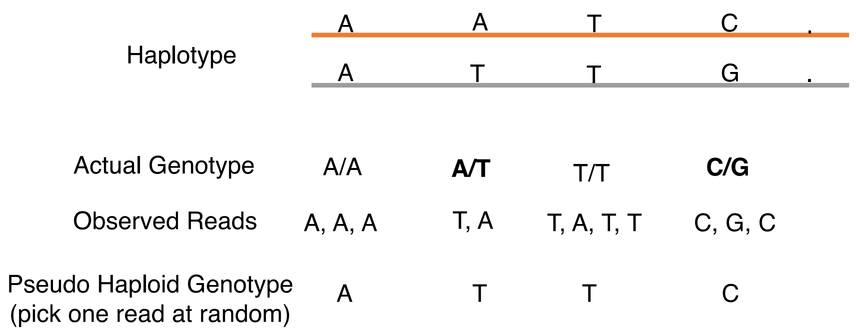

Supplementary Fig. 1: Pseudo haploidization scheme showing random allele calling for the generation of multi-locus genotypes.

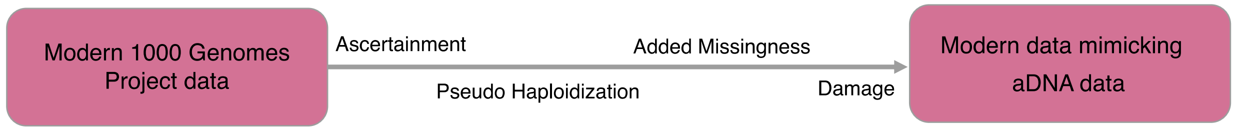

Supplementary Fig. 2: Data processing scheme, we take modern genomic data and apply ascertainment, pseudo-haploidization, add missingness, and incorporate damage to the data to make it mimic the artefacts of aDNA (ancient DNA) data used in this study.

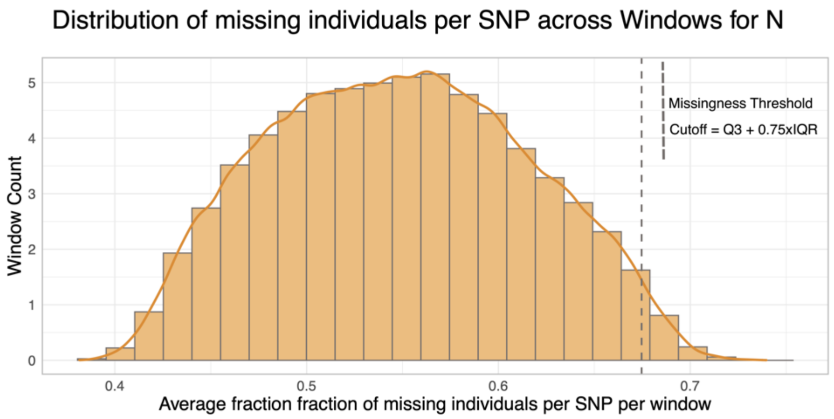
Distribution of missing individuals per SNP across Windows for N
Window Count
0
1
2
3
4
5
0.4
0.5
0.6
0.7
Missingness Threshold
Cutoff = Q3 + 0.75xIQR
Average fraction fraction of missing individuals per SNP per window

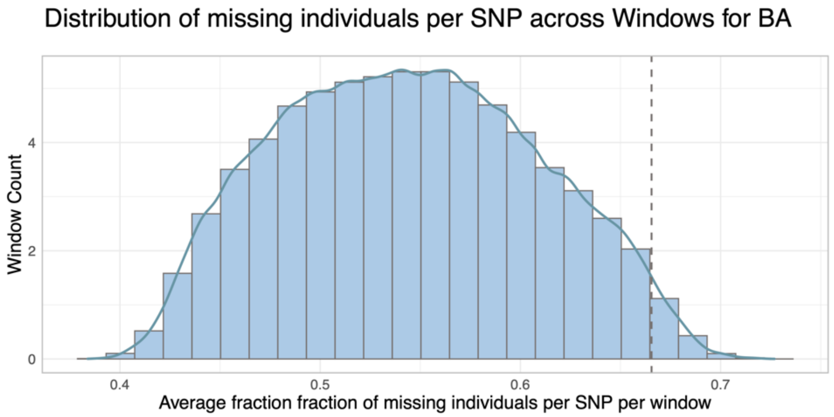
Distribution of missing individuals per SNP across Windows for BA
Window Count
0
2
4
0.4
0.5
0.6
0.7
Average fraction fraction of missing individuals per SNP per window

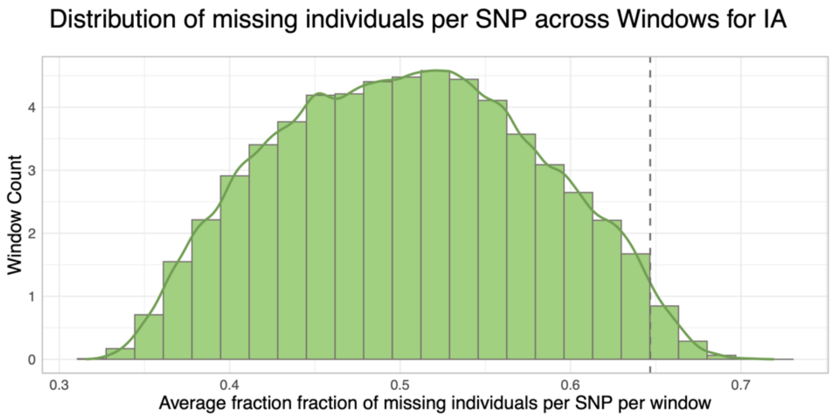
Distribution of missing individuals per SNP across Windows for IA
Window Count
0
1
2
3
4
0.3
0.4
0.5
0.6
0.7
Average fraction fraction of missing individuals per SNP per window

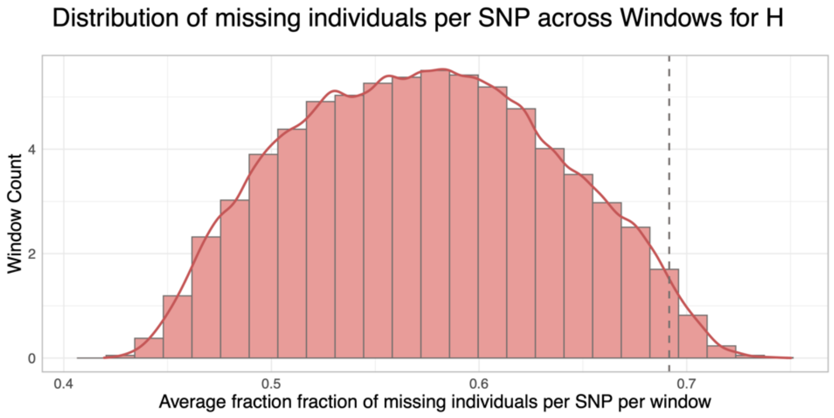
Distribution of missing individuals per SNP across Windows for H
Window Count
0
2
4
0.4
0.5
0.6
0.7
Average fraction fraction of missing individuals per SNP per window

Supplementary Fig. 3: Histograms of mean fraction of missing individuals per SNP per window. Here we show the histograms across epoch for the average fraction of missing individuals SNP per window genome wide. The vertical dashed line in each plot shows a genome wide cut off for excluding windows with high fraction of missing individuals, we calculated it using the mean fraction of missingness per SNP per window genome wide across SNPs and the cutoff can be represented as Q3 + 0.75×IQR (where Q3 is third quartile and IQR is inter quartile range).

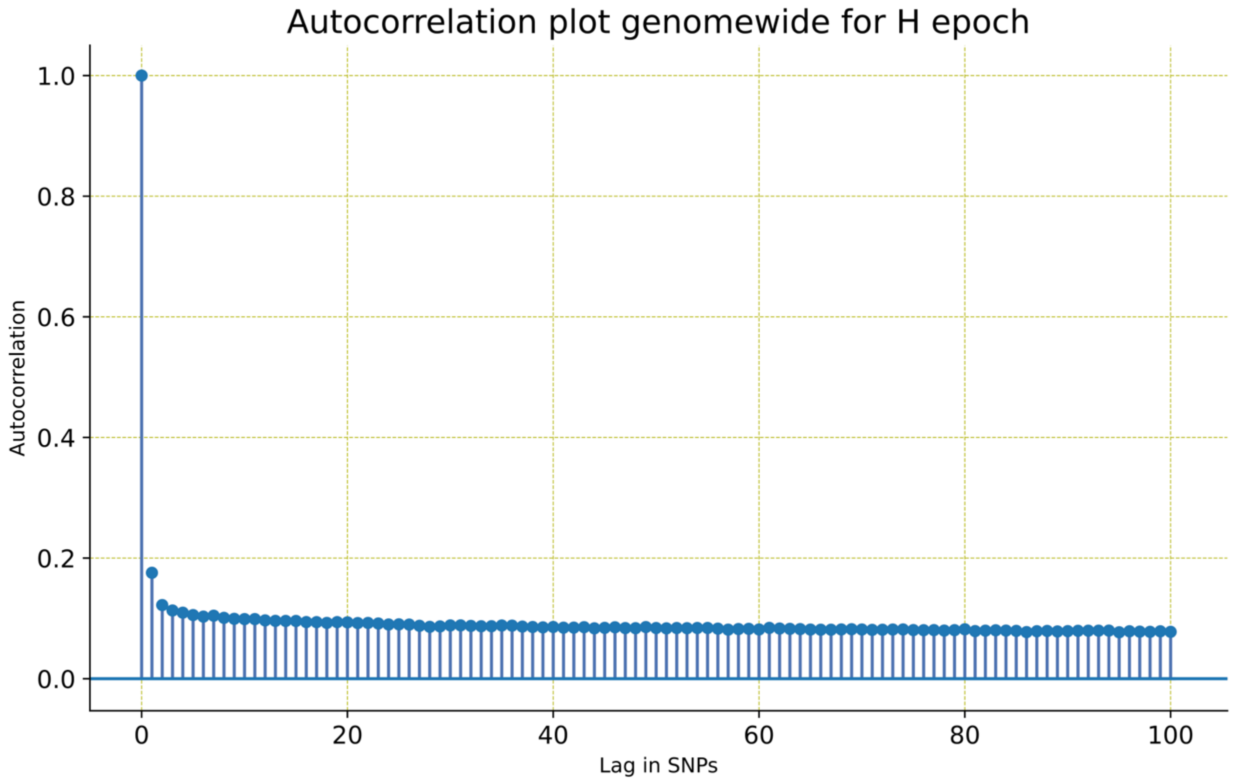

Supplementary Fig. 4: Plot showing the autocorrelation values for fraction of missing individuals between neighboring SNPs. After the lag of 2 SNPs the autocorrelation becomes almost constant ranging between 0.18 to 0.20. This implies that the missingness in the data is not region specific.

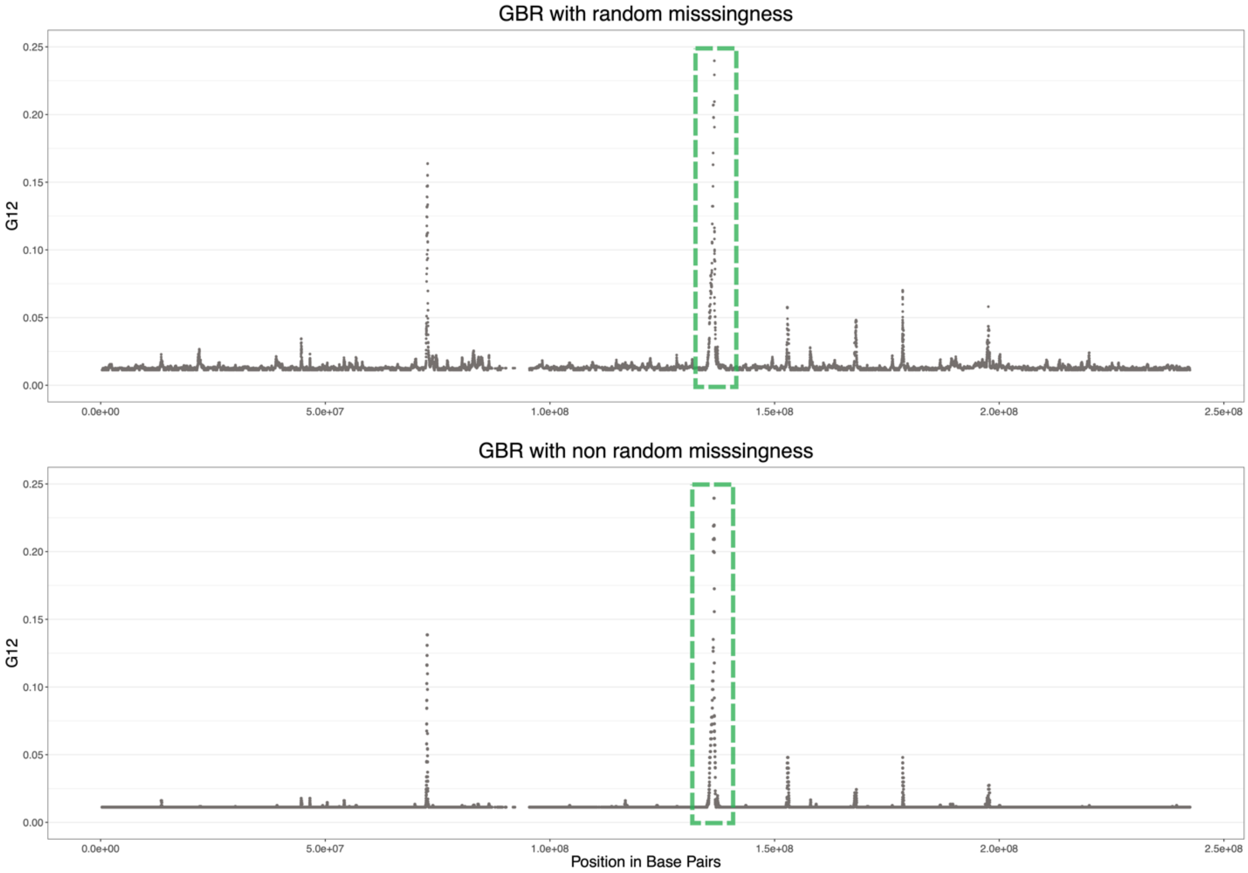

Supplementary Fig. 5: Comparison of G12 scans with random and non-random missingness. In both the scenarios we were able to recover *LCT* in GBR chromosome 2. It validates that G12 is robust to region specific and non-random missingness.

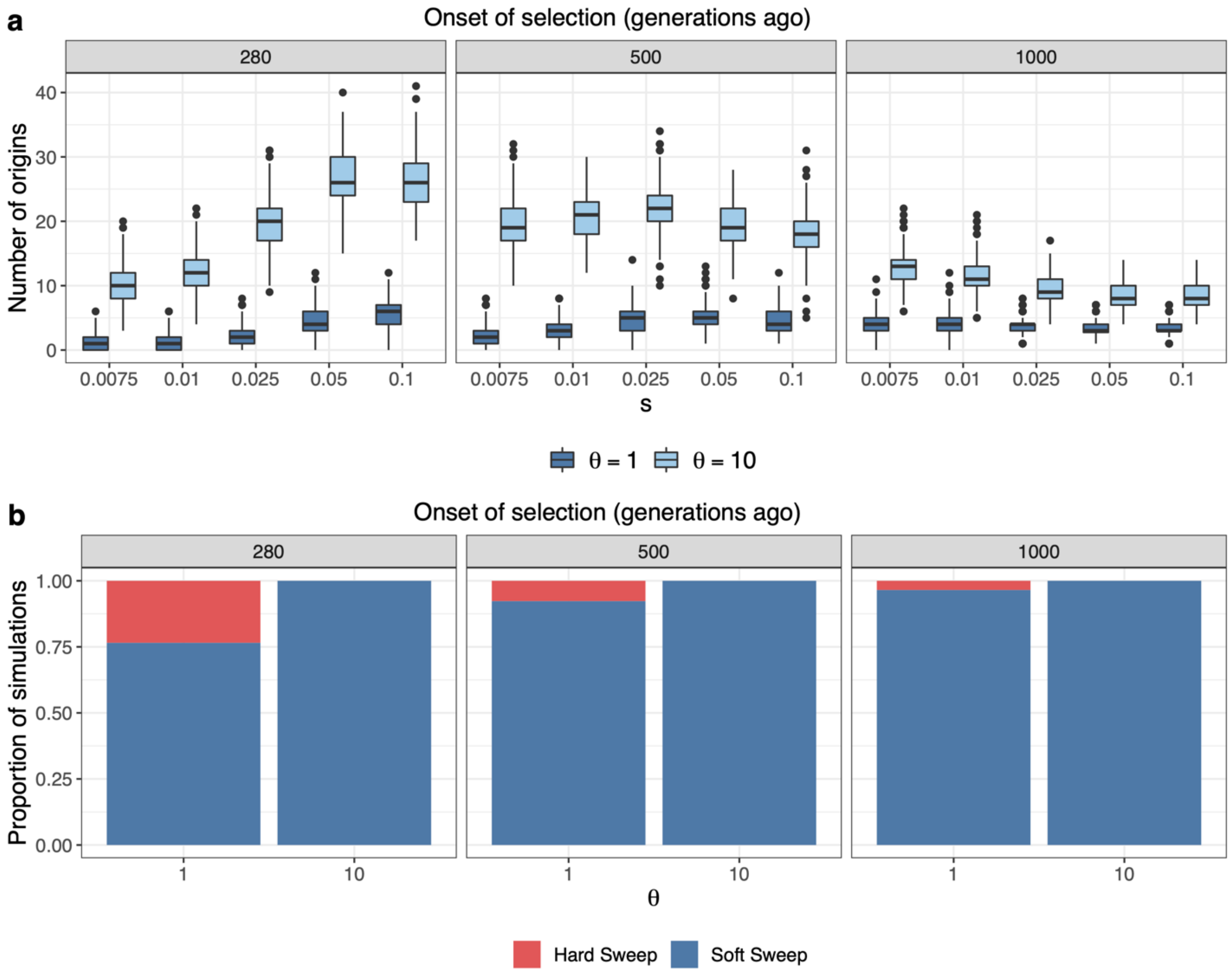

Supplementary Fig. 6. Softness of sweeps arising from recurrent de novo mutations. a Number of mutational origins for θ_A =1 and θ_A =10 for three different times of onset of selection. **b** Proportion of simulations with a single origin (red) and with two or more origins (blue). All simulations were sampled 40 generations before present.

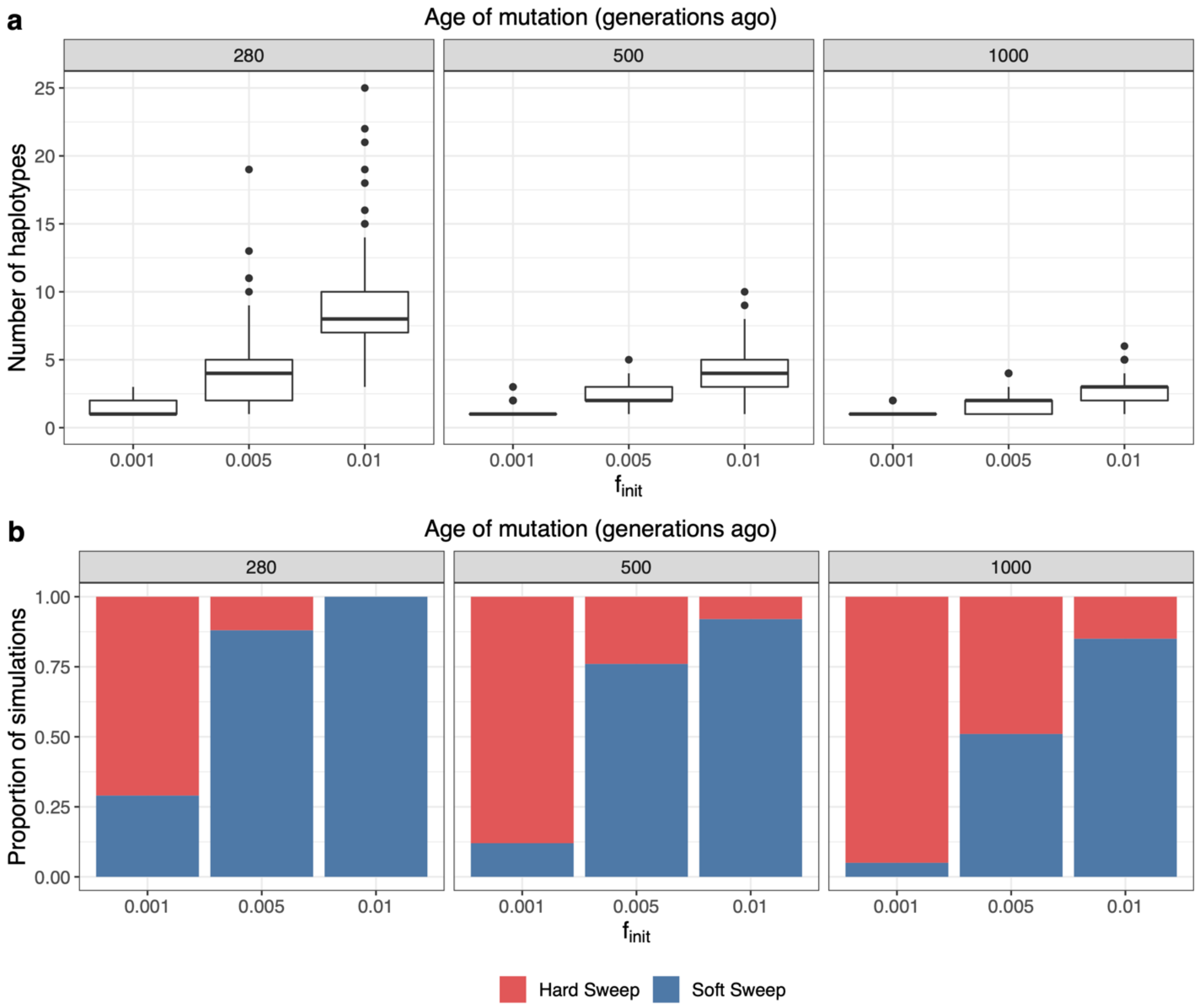

Supplementary Fig. 7. Softness of sweeps arising from SGV. a Number of distinct haplotypes bearing the adaptive mutation for three different ages of the mutation at the onset of selection. **b** Proportion of simulations with one haplotype (red) and with two or more haplotypes (blue) before the onset of selection. f_{init} is the initial allele frequencies.

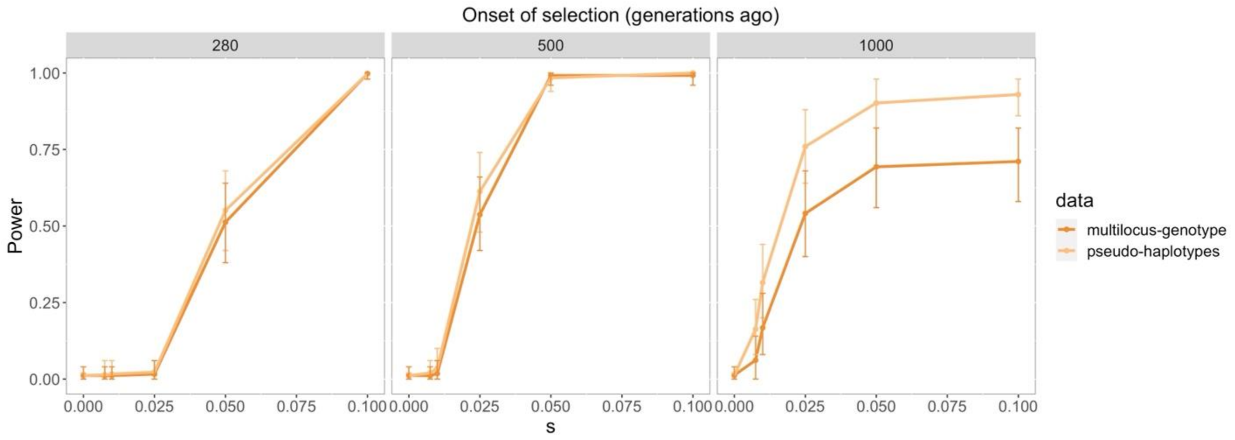

Supplementary Fig. 8: Power analysis for G12 computed on multi-locus genotypes versus pseudo-haplotypes. Selection started at 280, 500 and 1000 generations ago and 177 individuals were sampled 40 generations ago. No missing data was added to the simulated data. We ran a total of 500 hard sweep simulations for each combination of parameters with mutation rate μ= 1.25×10^{-8}/bp, chromosome length $L=5\times10^{5}$ and recombination $r = 1\times10^{-8}$ events/bp. Mean power and 95% confidence intervals measured at a 1% FDR are shown for increasing selection strengths. FDR: False Discovery Rate and s: selection coefficient.

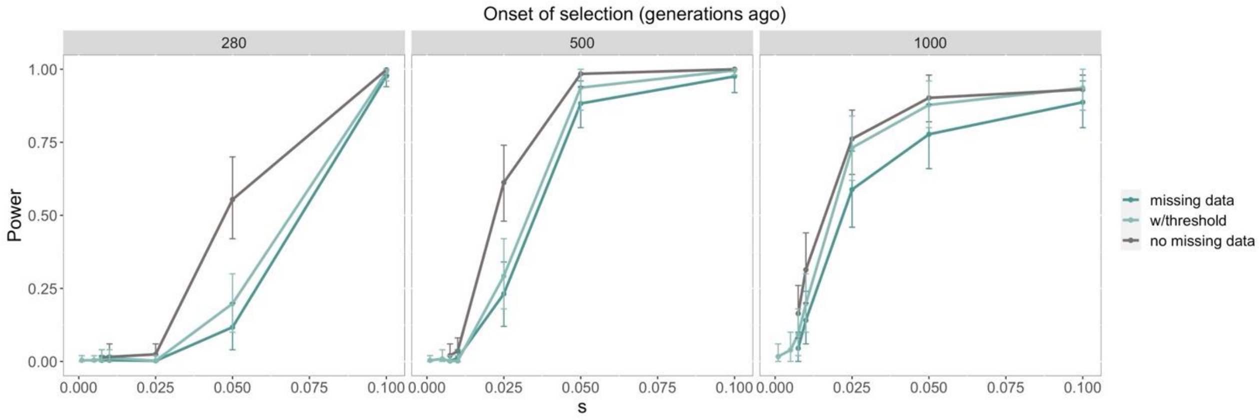

Supplementary Fig. 9: Power analysis for G12 computed on pseudo-haploidized simulated data with varying rates of missing data. Selection started at 280, 500 and 1000 generations ago and 177 individuals were sampled 40 generations ago. We compare G12 power on pseudo-haplotypes with and without missing data. Power is diminished when there is missing data. To improve power, we introduce a missingness threshold where if a haplotype has more than > 90% missing data, we do not cluster it with an existing haplotype group and instead treat it as a unique haplotype. In these simulations we introduce missing data with a mean rate of 0.55 missing data per SNP and a standard deviation of 0.23. Mean power and 95% confidence intervals measured at a 1% FDR are shown for increasing selection strengths. s: selection coefficient.

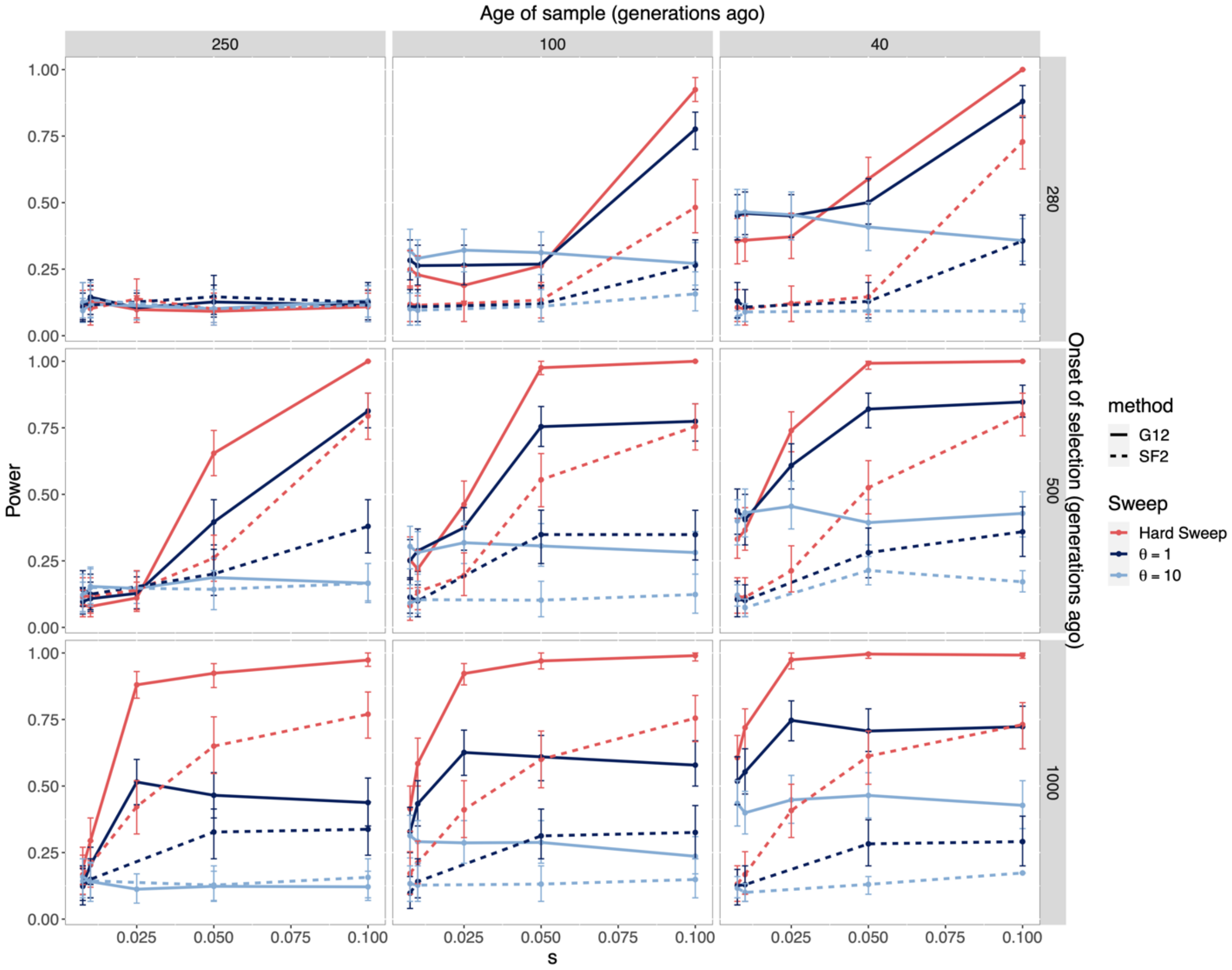

Supplementary Fig. 10: Power of G12 and SF2 in detecting hard (red) versus soft (blue) sweeps arising from recurrent *de novo* mutations in simulated aDNA (ancient DNA) data. Simulated aDNA included missing SNPs and pseudo-haplodization. We varied the selection strength of the sweeps (s), the onset of selection (rows) and sample generation (columns). We computed G12 and the SF2 CLR scores in a total of 2,000 simulations (500 hard sweeps, 500 sweeps from *de novo* for $\theta_A = 1$, 500 sweeps from *de novo* for $\theta_A = 1$, and 500 neutral simulation) for each combination of parameters with mutation rate $\mu = 1.25\times10^{-8}$ /bp, chromosome length $L=5\times10^{5}$ and recombination $r = 1\times10^{-8}$ events/bp. Mean power and 95% confidence intervals measured at a 1% FDR are shown for increasing selection strengths. s: selection coefficient.

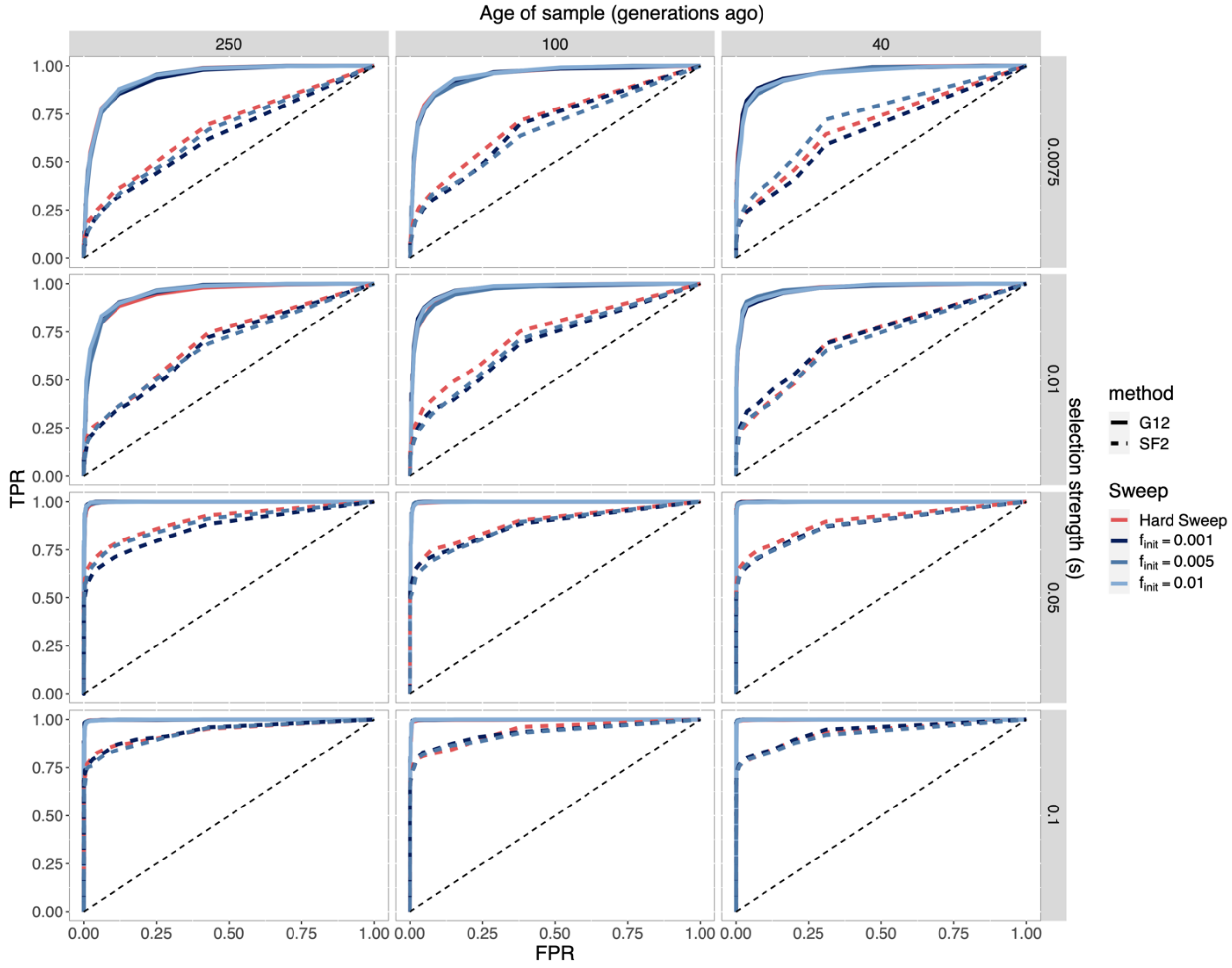

Supplementary Fig. 11: **Receiver operating characteristic (ROC) curves of G12 and SF2 in detecting single origin hard (red) versus SGV (blue) sweeps in simulated aDNA (ancient DNA) data**. Each panel shows ROC curves for G12 and SF2 for varying strengths of selection (rows) and age of sample (columns). For the simulations considered here, the onset of selection was set to 1000 generations before present. We computed G12 and the SF2 CLR scores in a total of 2,500 simulations (500 hard sweeps, 500 SGV sweeps with f_{init} = 0.001, 500 SGV sweeps with f_{init} = 0.005, 500 SGV sweeps with f_{init} = 0.01, and 500 neutral simulation) for each combination of parameters with mutation rate $\mu = 1.25\times10^{-8}$/bp, chromosome length $L = 5\times10^{5}$ and recombination $r = 1\times10^{-8}$ events/bp. TPR: True Positive Rate, FPR: False Positive Rate.

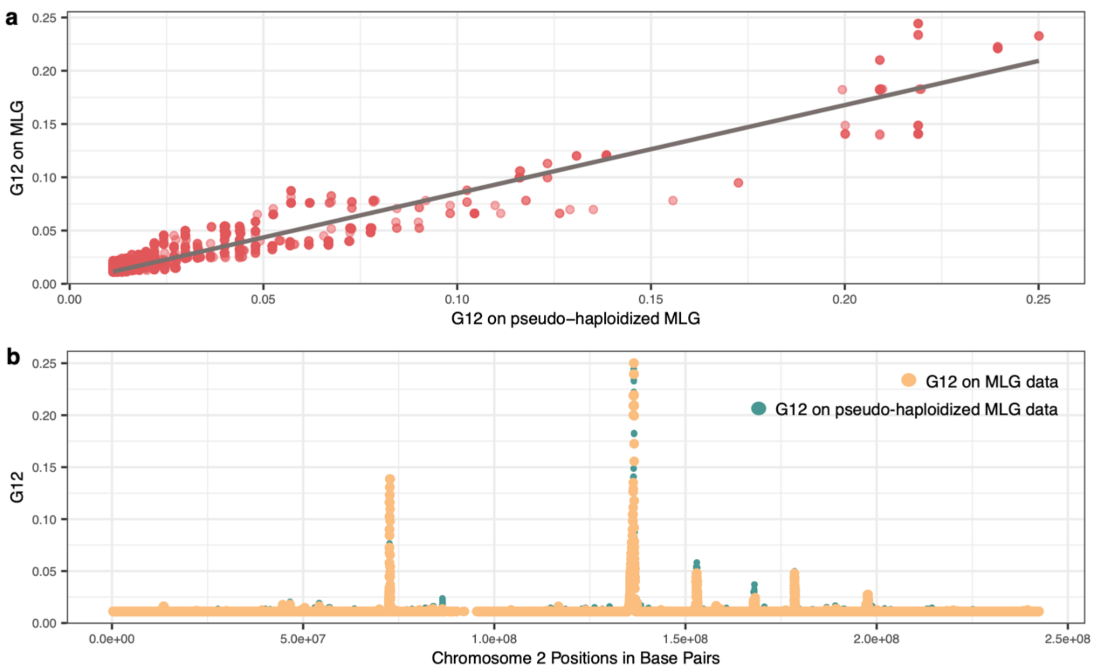

Supplementary Fig. 12: Plots showing strong positive correlation between G12 values on pseudo-haploidized Multilocus Genotype (MLG) vs diploid data for GBR individuals. **a** Scatter plot between G12 values for both scenarios with a line of best fit showing values are highly correlated. **b** Scatter plot between SNP positions and G12 values in both scenarios (pseudo haploidized and diploid) showing that both plots overlay each other to a very higher degree.

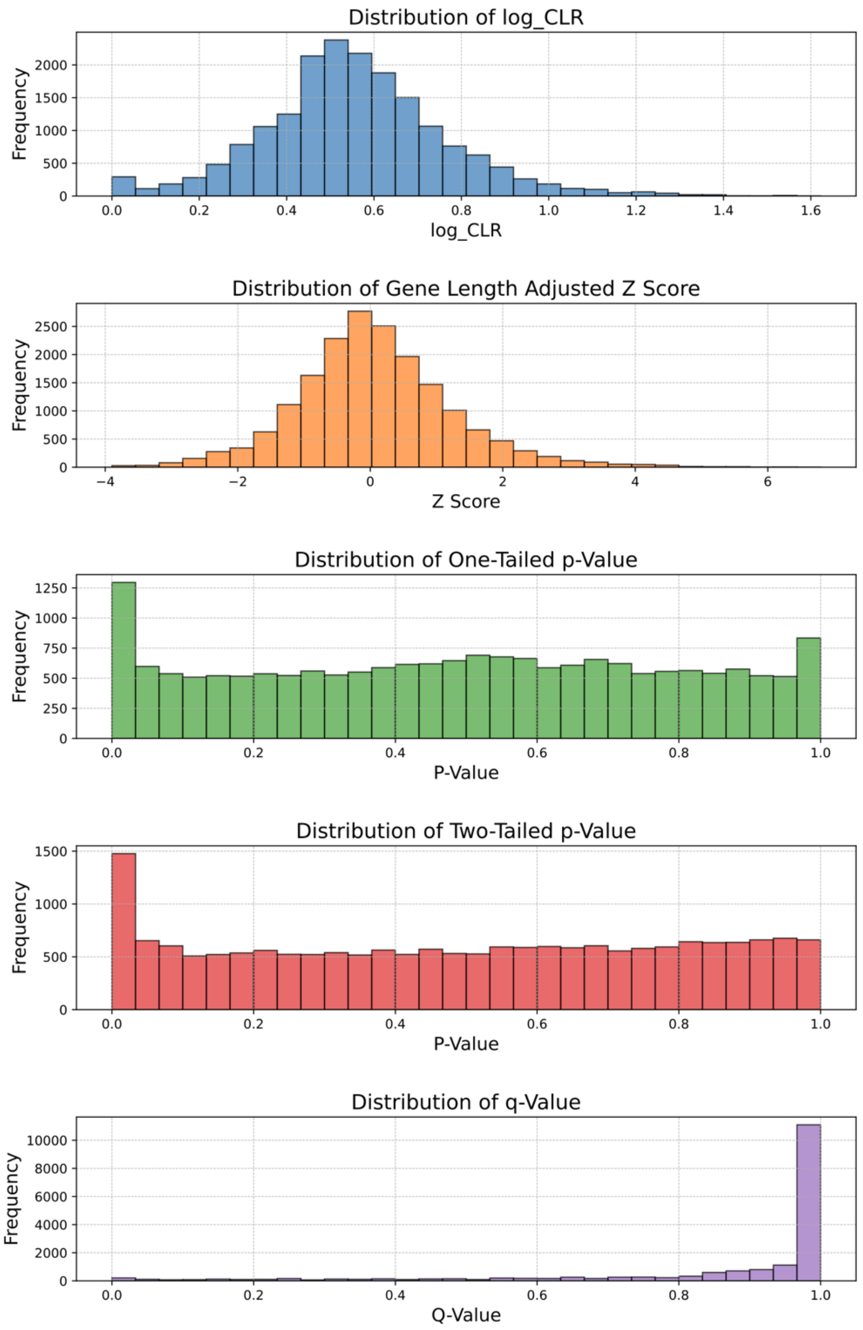

Supplementary Fig. 13: Distribution of all the transformations applied during outlier gene detection process. Gene length adjusted $log10$ transformed CLR scores are approximately standard gaussian and used as Z-score for further analysis. One tailed p values follow a U-shaped distribution, but two tailed p-values have J-shaped distribution which is one of the assumptions of q value correction.

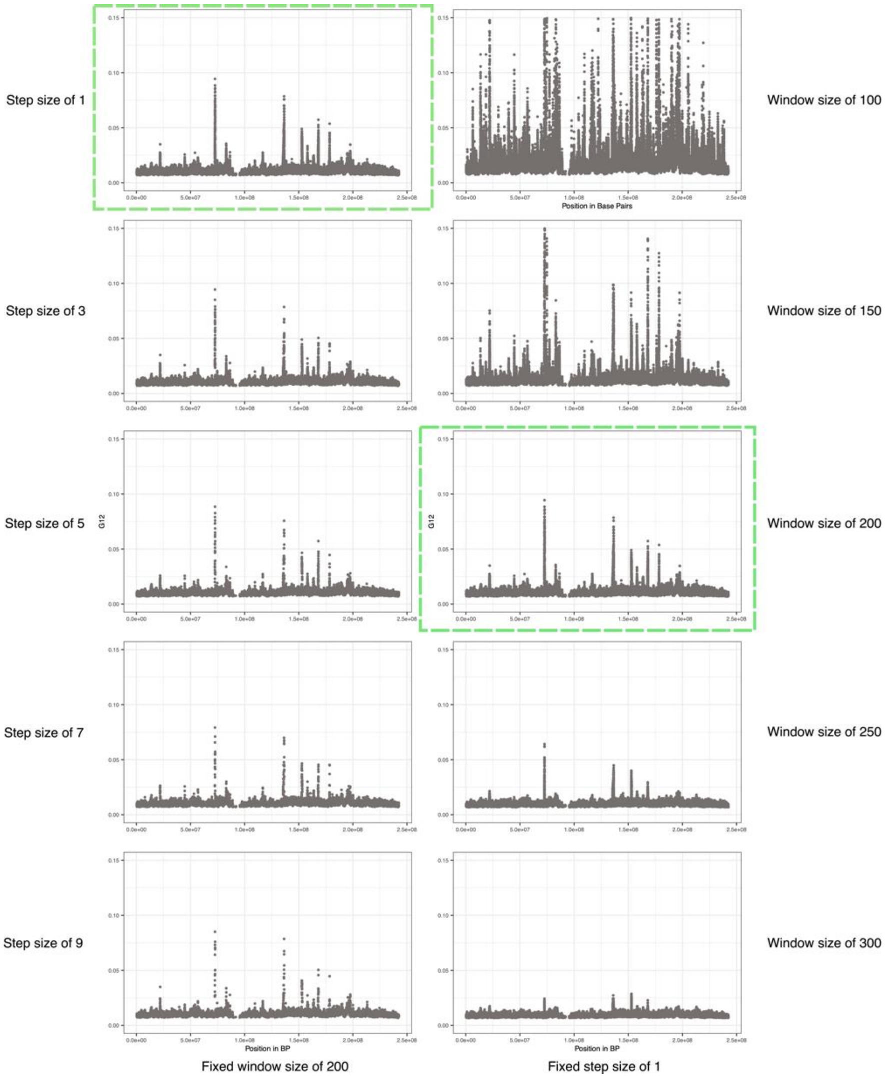

Supplementary Fig. 14: The performance of G12 selection scans on different window size and jump/step size values in terms of the number of SNPs. Left Panel: Variation of step/jump size (in terms of SNPs) while keeping the window size constant at 200. Right Panel: Variation of window size parameter (in terms of SNPs) while keeping jump/step size fixed at 1 (the parameter where noise was minimized in the Left Panel. In green boxes are our chosen optimal parameters.

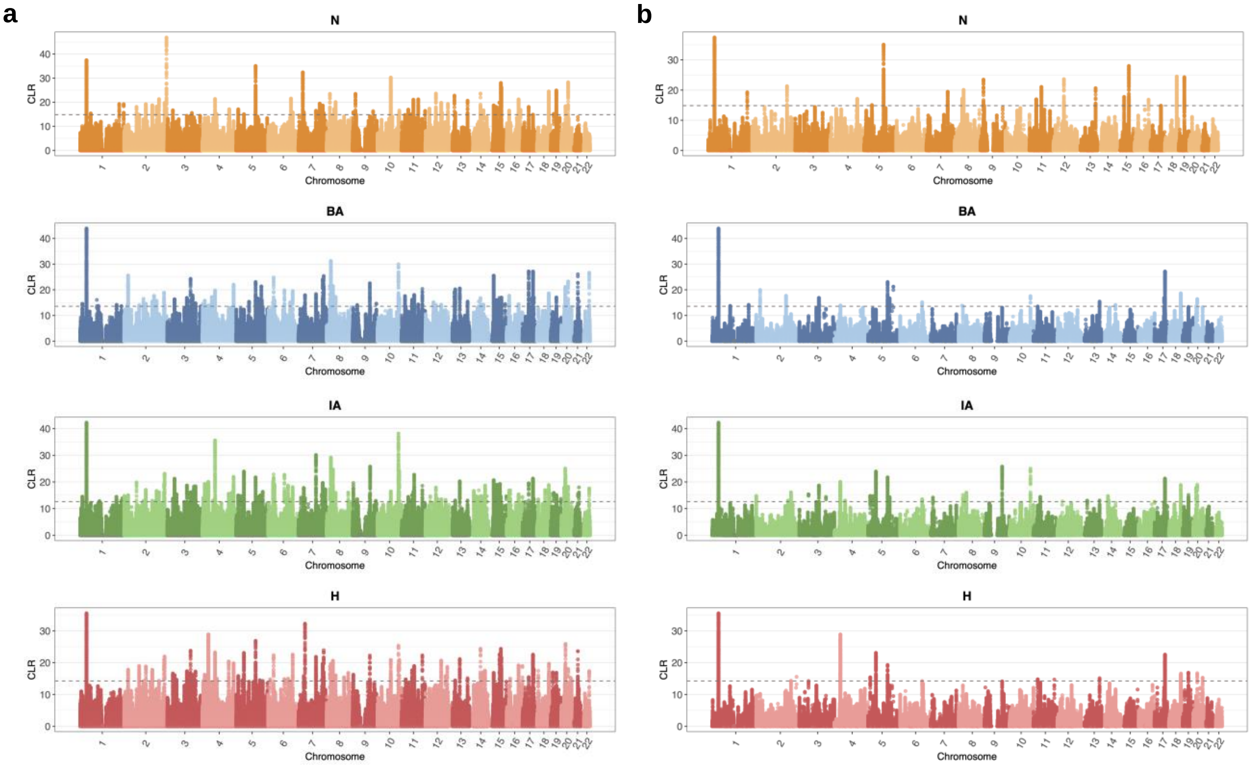

Supplementary Fig. 15: Comparison of SF2 output pre and post QC. **a** Manhattan plots showing the genome wide significant SF2 peaks for each epoch. The significance threshold (horizontal dashed line) is obtained as described in **Methods**: *Running selection scans on modern data mimicking aDNA*. There are a lot of significant peaks across epochs. **b** Manhattan plots post QC where positions lying in low recombination rate regions as well as high missingness regions have been removed. Post QC a large portion of sweeps disappear suggesting they were false positives. The section highlighted on chromosome 2 in the H epoch represents the region where we were unable to identify a selective signal at the *LCT* gene locus.

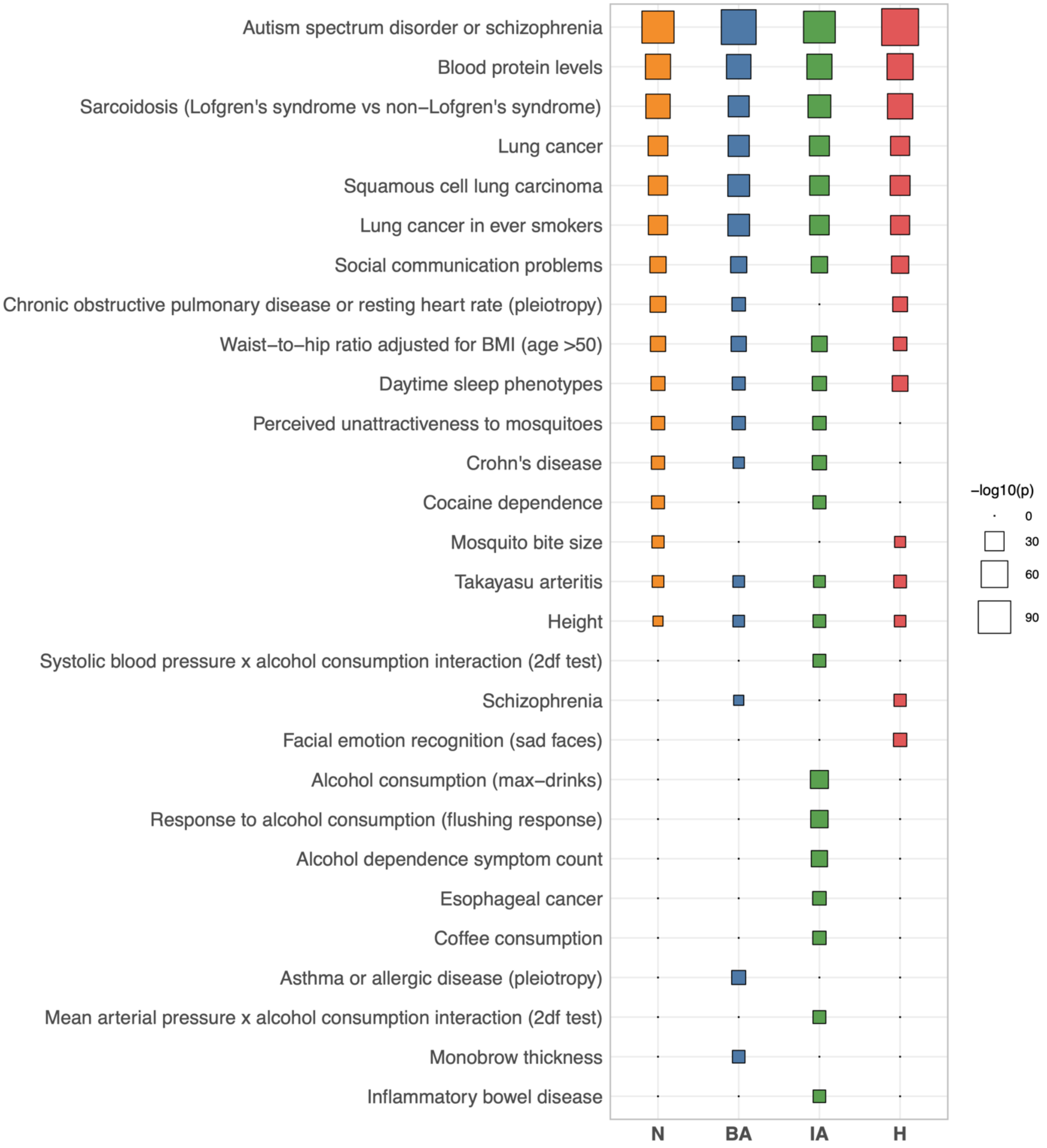

Supplementary Fig. 16: Gene sets enriched across epochs. Results of the enrichment analysis of selection signals overlapping loci seen in different association studies are shown along with significance in $log_{10}\,p$ values. Tests that were not significant are shown with a dot.

Parameter	Modern Samples	Ancient Samples
Mean Missingness (Preprocessing)	0.0131	0.54827
Mean Missingness (Post-processing)	0.53529	0.54827

Supplementary Table 1: Differences between the mean fraction of missing individuals per SNP in modern samples vs. the ancient samples, pre, and post-data processing.

Gene	Population	Chr	Position	Function
SLC24A5	CEU	15	Band: 15q21.1<br>Start: 48,120,990 bp<br>End: 48,142,672 bp	This locus is one of the major factors influencing skin pigmentation in humans
LCT/MCM6	CEU	2	Band 2q21.3<br>Start 135,839,626 bp<br>End 135,876,443 bp	This enzyme helps to digest lactose, a sugar found in milk and other dairy products
TLR1	CEU	4	Band 4p14<br>Start 38,790,677 bp<br>End 38,856,817 bp	Toll-like receptors are a class of proteins that play a key role in the innate immune system

Supplementary Table 2: The variants of interest that are shown to be under selection by multiple natural selection studies on European genomes.

Epoch	ND across 200 SNP window	Total number of sites	Segregating sites (S)	S/BP	Mean Window Length (bp)
N	0.00002752173	1233013	930906	0.754984	454774 (± 386740)
BA	0.00002817481	1233013	953594	0.773385	454507 (± 386870)
IA	0.00004058848	1233013	962723	0.780789	455005 (± 386849)
H	0.00004152787	1233013	943001	0.764794	454605 (± 386845)

Supplementary Table 3: A table showing the nucleotide diversity (ND) calculated for each epoch on a 200 SNP window. We used the vcftools --window-pi option which measures the nucleotide diversity in windows. We also show the number of segregating sites per base pair.

**Variable**	**G12**	
	R^2	Correlation Coefficient
Window Size	0.013	0.1030
Recombination Rate	0.001	-0.0261
Missingness	0.027	0.1634

Supplementary Table 4: Relationship between parameter choice and G12 value suggests that overall G12 statistics are unaffected by our choice of parameters.
