# Supplementary material for: Leveraging ancient DNA to uncover signals of natural selection in Europe lost due to admixture or drift
Source: Nat Commun. 2024 Nov 12;15:9772. doi: 10.1038/s41467-024-53852-8 (PMC11557891; doi:10.1038/s41467-024-53852-8)
Supplement: Supplementary file 2 — Description of Additional Supplementary Files [file 41467_2024_53852_MOESM2_ESM.pdf]

Description of Additional Supplementary Files

File Name: Supplementary Data 1

Description: This file contains multiple sheets providing supplementary data related to the study. Below is a description of each sheet.

- **Sheet 1: List of ancient samples**
  Description: Contains information on ancient samples, including Sample ID, Epoch, Master ID, skeletal code, skeletal element, and publication details. Data spans various epochs and includes metadata on sample publication history.
- **Sheet 2: Genes mapped to G12 peaks**
  Description: Lists genes that are mapped to significant G12 peaks identified in the study. Includes chromosome positions and corresponding gene information.
- **Sheet 3: CEU SF2 outlier genes Pre-QC**
  Description: Contains a list of outlier genes in the CEU population based on SweepFinder2 (SF2) before quality control. Includes chromosome positions and gene annotations.
- **Sheet 4: YRI SF2 outlier genes Pre-QC**
  Description: Contains a list of outlier genes in the YRI population based on SweepFinder2 before quality control. Includes chromosome positions and gene annotations.
- **Sheet 5: STU SF2 outlier genes Pre-QC**
  Description: Contains a list of outlier genes in the STU population based on SweepFinder2 before quality control. Includes chromosome positions and gene annotations.
- **Sheet 6: N SF2 outlier genes Pre-QC**
  Description: Contains a list of outlier genes in the Neolithic (N) population based on SweepFinder2 before quality control. Includes chromosome positions and gene annotations.
- **Sheet 7: N SF2 outlier genes Post-QC**
  Description: Contains a list of outlier genes in the Neolithic (N) population based on SweepFinder2 after quality control. Includes chromosome positions and gene annotations.
- **Sheet 8: BA SF2 outlier genes Pre-QC**
  Description: Contains a list of outlier genes in the Bronze Age (BA) population based on SweepFinder2 before quality control. Includes chromosome positions and gene annotations.
- **Sheet 9: BA SF2 outlier genes Post-QC**
  Description: Contains a list of outlier genes in the Bronze Age (BA) population based on SweepFinder2 after quality control. Includes chromosome positions and gene annotations.
- **Sheet 10: IA SF2 outlier genes Pre-QC**
  Description: Contains a list of outlier genes in the Iron Age (IA) population based on SweepFinder2 before quality control. Includes chromosome positions and gene annotations.

- **Sheet 11: IA SF2 outlier genes Post-QC**
  Description: Contains a list of outlier genes in the Iron Age (IA) population based on SweepFinder2 after quality control. Includes chromosome positions and gene annotations.
- **Sheet 12: H SF2 outlier genes Pre-QC**
  Description: Contains a list of outlier genes in the Historical (H) population based on SweepFinder2 before quality control. Includes chromosome positions and gene annotations.
- **Sheet 13: H SF2 outlier genes Post-QC**
  Description: Contains a list of outlier genes in the Historical (H) population based on SweepFinder2 after quality control. Includes chromosome positions and gene annotations.
