# Supplementary material for: Leveraging ancient DNA to uncover signals of natural selection in Europe lost due to admixture or drift
Source: Nat Commun. 2024 Nov 12;15:9772. doi: 10.1038/s41467-024-53852-8 (PMC11557891; doi:10.1038/s41467-024-53852-8)
Supplement: Supplementary file 4 — Reporting Summary [file 41467_2024_53852_MOESM4_ESM.pdf]

nature portfolio

Corresponding author(s): Nandita R Garud and Vagheesh Narasimhan

Last updated by author(s): 10/04/2024

Reporting Summary

Nature Portfolio wishes to improve the reproducibility of the work that we publish. This form provides structure for consistency and transparency in reporting. For further information on Nature Portfolio policies, see our Editorial Policies and the Editorial Policy Checklist.

Statistics

For all statistical analyses, confirm that the following items are present in the figure legend, table legend, main text, or Methods section.

n/a	Confirmed	
☐	☒	The exact sample size (*n*) for each experimental group/condition, given as a discrete number and unit of measurement
☐	☒	A statement on whether measurements were taken from distinct samples or whether the same sample was measured repeatedly
☐	☒	The statistical test(s) used AND whether they are one- or two-sided<br>*Only common tests should be described solely by name; describe more complex techniques in the Methods section.*
☐	☒	A description of all covariates tested
☐	☒	A description of any assumptions or corrections, such as tests of normality and adjustment for multiple comparisons
☐	☒	A full description of the statistical parameters including central tendency (e.g. means) or other basic estimates (e.g. regression coefficient) AND variation (e.g. standard deviation) or associated estimates of uncertainty (e.g. confidence intervals)
☐	☒	For null hypothesis testing, the test statistic (e.g. *F*, *t*, *r*) with confidence intervals, effect sizes, degrees of freedom and *P* value noted<br>*Give P values as exact values whenever suitable.*
☒	☐	For Bayesian analysis, information on the choice of priors and Markov chain Monte Carlo settings
☒	☐	For hierarchical and complex designs, identification of the appropriate level for tests and full reporting of outcomes
☐	☒	Estimates of effect sizes (e.g. Cohen's *d*, Pearson's *r*), indicating how they were calculated

Our web collection on statistics for biologists contains articles on many of the points above.

Software and code

Policy information about availability of computer code

Data collection	We did not use any open source or custom code to collect data for this study. We collected the data from Allen Ancient DNA Resource (AADR). The specifics of which are available in this recent pre-print: Mallick, S. et al. The Allen Ancient DNA Resource (AADR): A curated compendium of ancient human genomes. (2023) doi:10.1101/2023.04.06.535797. The data we used is from AADR version 51 and can be accessed here: https://reich.hms.harvard.edu/allen-ancient-dna-resource-aadr-downloadable-genotypes-present-day-and-ancient-dna-data
Data analysis	We wrote custom code for our study. The code used for doing simulation studies could be found here: https://github.com/mariharris/G12ancient_simulations and the code used for performing selection scans can be found here: https://github.com/ngarud/SelectionHapStats

For manuscripts utilizing custom algorithms or software that are central to the research but not yet described in published literature, software must be made available to editors and reviewers. We strongly encourage code deposition in a community repository (e.g. GitHub). See the Nature Portfolio guidelines for submitting code & software for further information.

Data

Policy information about availability of data

All manuscripts must include a data availability statement. This statement should provide the following information, where applicable:

- Accession codes, unique identifiers, or web links for publicly available datasets
- A description of any restrictions on data availability
- For clinical datasets or third party data, please ensure that the statement adheres to our policy

: Code used for running G12ancient selection scans can be found here: https://github.com/ngarud/SelectionHapStats, Code for running simulations can be found here: https://github.com/mariharris/Ancient_DNA_simulations . The ancient genomes used in this work can be accessed at Allen Ancient DNA Resource (AADR), version 51: https://reich.hms.harvard.edu/allen-ancient-dna-resource-aadr-downloadable-genotypes-present-day-and-ancient-dna-data

Research involving human participants, their data, or biological material

Policy information about studies with human participants or human data. See also policy information about sex, gender (identity/presentation), and sexual orientation and race, ethnicity and racism.

Reporting on sex and gender	Our research only examines ancient genetic sequences and this does not apply.
Reporting on race, ethnicity, or other socially relevant groupings	Our research only examines ancient genetic sequences and this does not apply.
Population characteristics	Our research only examines ancient genetic sequences and this does not apply.
Recruitment	Our research only examines ancient genetic sequences and this does not apply.
Ethics oversight	Our research only examines ancient genetic sequences and this does not apply.

Note that full information on the approval of the study protocol must also be provided in the manuscript.

Field-specific reporting

Please select the one below that is the best fit for your research. If you are not sure, read the appropriate sections before making your selection.

☐ Life sciences ☐ Behavioural & social sciences ☒ Ecological, evolutionary & environmental sciences

For a reference copy of the document with all sections, see nature.com/documents/nr-reporting-summary-flat.pdf

Ecological, evolutionary & environmental sciences study design

All studies must disclose on these points even when the disclosure is negative.

Study description	Here we have performed natural selection study on populations from Holocene Europe using multi-locus genotype identity scans. We have validated our analysis using simulations, positive and, negative control variants.
Research sample	We used a total sample size of 708 ancient individuals. We collected these samples from AADR version 51. To minimize reference bias or batch effects associated with data processing issues across the set of samples, we chose to include only samples for which hybridization capture was performed on 1.2 million positions and that had at least 15,000 SNPs for which we could perform high-quality population genetic analysis.
Sampling strategy	We have samples from 4 different time periods, to maintain homogeneity across our analysis we have used a fixed sample size of 177 ancient individuals for each time period, which resulted in a final sample size of 708 individuals.
Data collection	The data is collected from AADR, version 51 and can be accessed here: https://reich.hms.harvard.edu/allen-ancient-dna-resource-aadr-downloadable-genotypes-present-day-and-ancient-dna-data
Timing and spatial scale	In our analysis we examined a collection of 708 recently published samples from Europe ranging from 6572 BP to 353 BP. All samples were from Europe.
Data exclusions	No data were excluded from analysis
Reproducibility	All code and data are fully available for reproducibility. All samples available were used in the primary analysis.
Randomization	Our analysis does not require data randomization.

Blinding	Our analysis does not require blinding.

Did the study involve field work? ☐ Yes ☒ No

Reporting for specific materials, systems and methods

We require information from authors about some types of materials, experimental systems and methods used in many studies. Here, indicate whether each material, system or method listed is relevant to your study. If you are not sure if a list item applies to your research, read the appropriate section before selecting a response.

Materials & experimental systems

n/a	Involved in the study
☒	☐ Antibodies
☒	☐ Eukaryotic cell lines
☒	☐ Palaeontology and archaeology
☒	☐ Animals and other organisms
☒	☐ Clinical data
☒	☐ Dual use research of concern
☒	☐ Plants

Methods

n/a	Involved in the study
☒	☐ ChIP-seq
☒	☐ Flow cytometry
☒	☐ MRI-based neuroimaging
